# Influences on food supply from elk abundance and precipitation early in the growing season

**DOI:** 10.1371/journal.pone.0264941

**Published:** 2022-03-11

**Authors:** Lee H. Williamson, Floyd W. Weckerly

**Affiliations:** Department of Biology, Texas State University, San Marcos, Texas, United States of America; Universitat Autonoma de Barcelona, SPAIN

## Abstract

Large grazing mammals should negatively affect forage biomass of their food supply, but documentation is lacking in free ranging populations. Furthermore, complications from factors such as weather patterns and spatial heterogeneity might obscure grazing effects on the food supply. We examined influences of Roosevelt elk (*Cervus canadensis roosevelti* (Merriam, 1897)) abundance and precipitation on forage biomass at two spatial scales; meadows that contained most of the food supply, and sectors nested in meadows. Spatial heterogeneity in forage biomass might also decline with increasing elk abundance. Elk abundance was estimated from population counts and varied 3.9-fold across the 15 years of study in northwestern California, USA. Each January, early in the growing season, we estimated forage biomass in the 50-ha meadow complex used by the elk population. Measures of palatable forage cover and height were taken in 270 ¼ m^2^ plots dispersed throughout sectors. These measurements were then related to dried forage biomass. At both spatial scales, elk abundance was inversely, and precipitation was positively related to forage biomass. At the sector scale, analysis of a linear mixed effect model indicated heterogeneity. In some sectors both predictors were related to forage biomass and in other sectors they were not. Heterogeneity was not from uneven elk grazing as elk grazed sectors in proportion to forage biomass. The varied elk abundance–forage biomass relationships across sectors indicated that spatial heterogeneity declined with increasing elk abundance. Detecting relationships between free ranging ungulate populations and biomass of their food supply is not straightforward.

## Introduction

Foraging by large herbivores should bring about a decline in the food supply when consumer abundance increases, particularly in populations with more bottom-up than top-down influences [1,2]. There is, however, a lack of studies examining free ranging ungulate populations where the biomass of the food supply is measured across low to high abundance where high abundance is when herbivore population growth is slowed as the population nears *K* carrying capacity. Perhaps one reason why the food supply has not been measured is the logistical challenges of directly measuring forage biomass. Also, there might be little motivation for investigation because density dependence is pervasive in large herbivore populations, and as such a decline in the food supply with increasing herbivore abundance is intuitive [1–3]. Nevertheless, the dynamics between communities of producers and consumer abundance might be complicated by herbivore optimization, weather, and patterns of plant growth at small spatial scales that might affect biomass of the food supply at the spatial scale occupied by the consumer population. These factors might complicate consumer–producer relationships and, in turn, impact ecosystem processes [4].

Grazing and trampling by large herbivores immediately decreases aboveground plant material especially in plant communities comprised of herbaceous, grazing-tolerant plants [5–7]. These disturbances, however, can create conditions that facilitate plant growth and even compensatory growth, particularly at low herbivore abundances [8–10]. Because large herbivores also trample vegetation when grazing, overstory senescent plant material is removed which increases the amount of sunlight that reaches new growth [11,12]. Additionally, the loss of plant material stimulates photosynthetic activity in remaining leaves [13] and can prompt physiological responses that promote leaf development [14]. These responses can allow the plant to elevate growth or compensate for and even benefit from herbivore disturbances, what is called herbivore optimization [8,10]. Herbivore optimization might make it difficult to detect an association between herbivore abundance and state of the food supply or forage biomass at low to moderate consumer abundances.

Plant growth in response to herbivore disturbances is also governed by available moisture [8,15]. Allocating resources towards above-ground primary production requires water to be diverted from the plants root system [16], and this can limit root development [15–17]. In areas with abundant moisture, however, plants can increase above ground primary production without compromising root development [15]. In plant communities comprised of grazing-tolerant plants, both moisture and herbivore disturbances should affect forage biomass [9,10].

Another dimension is the soil. Plant response to both moisture and herbivore disturbances is mediated by soil characteristics [9,10]. In areas with productive soils plant response to precipitation and herbivore disturbance is greater than in areas with less productive soils. Furthermore, the spatial scale at which soil characteristics vary can be as small as several meters [18]. When considering that ungulate populations typically range over spatial scales of kilometers, heterogeneity in plant response to precipitation and herbivore disturbance should be expected. The biomass of the food supply at the spatial scale inhabited by the ungulate population must then emerge from plant responses to precipitation and ungulate disturbances at smaller spatial scales.

In our study area in a temperate rainforest ecosystem, the forage habitat, meadows, are dominated by a single large herbivore, the Roosevelt elk (*Cervus canadensis roosevelti* (Merriam, 1897)). Compared to most populations of Roosevelt elk, the herds in our study area forages within a relatively small area [19]. This area consists of a simple matrix of dense forests with little forage intermixed with meadow forage patches. This setting makes it feasible to reliably estimate the bulk of the food supply. Across the 15 years of our study elk abundance varied 3.9-fold and density dependent population dynamics were evident, implying negative feedbacks between elk abundance and the food supply [19–21]. Herein, we measured elk abundance, precipitation, and forage biomass at two spatial scales across 15 years in winter which is early in the growing season in this temperate rainforest. In winter elk mostly graze grass and some forbs, and, furthermore, grazed parts of meadows in proportion to forage biomass [19,22]. Moreover, plants are especially responsive to moisture early in plant development which affects growth trajectories through the remainder of the growing season [9,21,23–27]. We tested the following expectations. One, at the spatial scale inhabited by an elk population, the meadow complex, the food supply should be inversely related to elk abundance and positively related to precipitation. The entire food supply should be affected by these factors because of patterns manifested at a smaller spatial scale. Two, at the smaller spatial scale of parts of the meadow complex, sectors, there should be a heterogeneous response in forage biomass to elk abundance and precipitation. In some sectors we expected forage biomass to respond to elk abundance and precipitation as expected at the large spatial scale. In other sectors we expected little if any detectable influence from abundance and precipitation on forage biomass. The heterogeneous, spatial response presumably reflects soil characteristics that were either amenable or unamenable to productive plant growth. Three, sector heterogeneity in forage biomass should decline with increasing elk abundance. The decline in sector or spatial heterogeneity in forage biomass should manifest from heterogeneous forage biomass response to elk abundance and precipitation across sectors. An inverse relationship between spatial heterogeneity in plant communities and ungulate grazing has been inferred in several ecosystems [28–30]. As far as we know, our study is the first to reveal some of the complications in uncovering inverse relationships between a free ranging, large grazing herbivore and estimates of its forage biomass.

## Methods

### Ethics statement

No animals were handled or approached to inter with and disrupt animal activity. Nonetheless, all animal research was reviewed and approved by the Texas State University Institutional Animal Care and Use Committee (IACUC), permit number 04-046876343F, 07-1106-07, 1035_1112_31, 1019_1031_23, 20168174611, 6839.

### Study area

**Fig 1:**
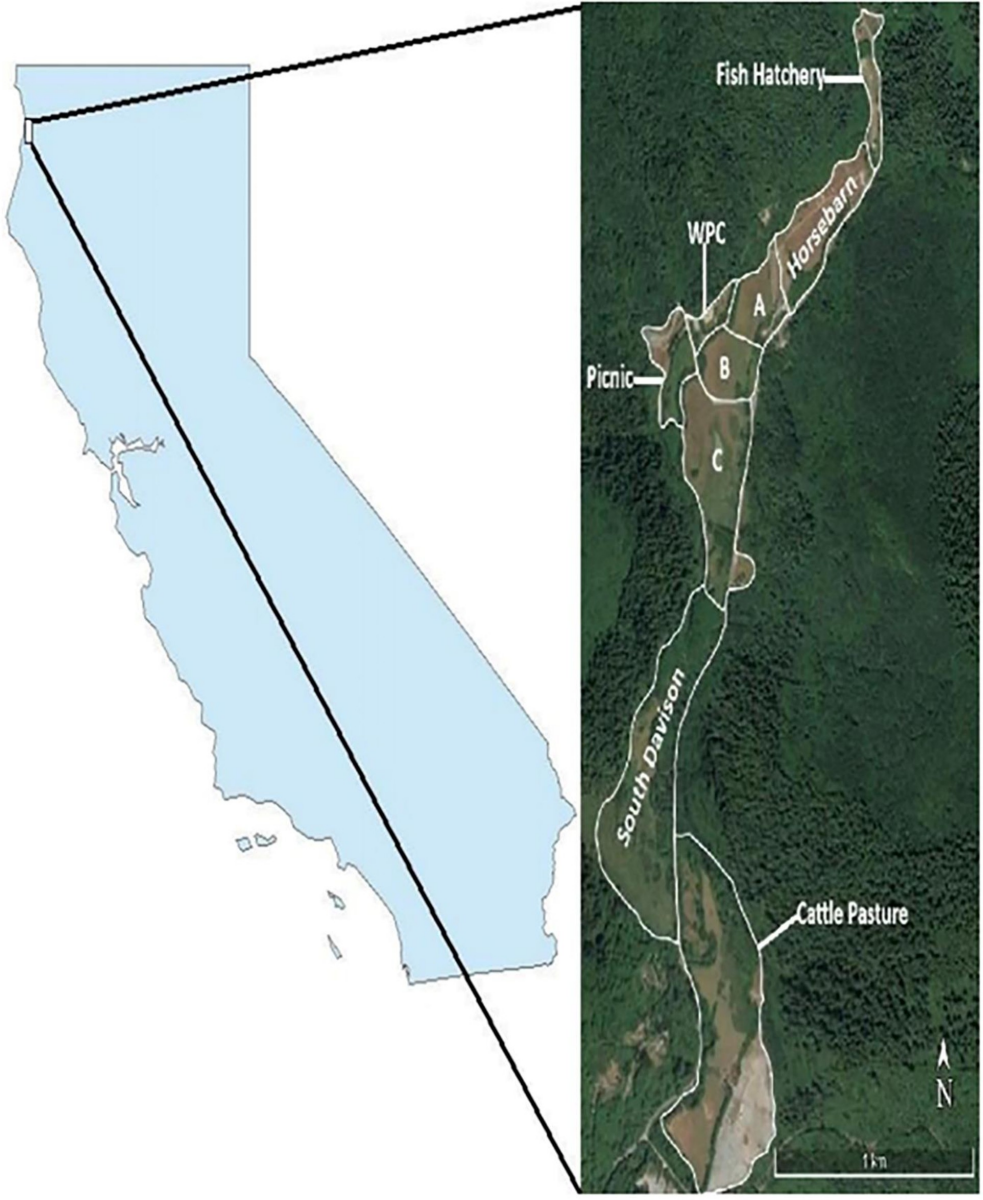
Map of the Davison meadow complex in Redwood National and State Parks and location of the parks in California. Within the meadow complex the sectors are delineated and named. The areal image was obtained from the National Agriculture Imagery Program (NAIP) from the USDA Farm Services Agency, is in the public domain, and has not been copyrighted to our knowledge.

Data was collected in the Davison meadow complex along the Prairie Creek drainage in Redwood National and State Parks (41 ^o^24’ N, 124^o^02’W) in Humboldt County, California, USA (Fig 1). This area is a temperate rainforest that has a maritime climate with temperatures that typically range 10–20°C year-round [21]. On average, the area receives 165 cm of precipitation, most of which occurs from late fall through early spring [21]. Vegetation in the Davison meadow complex is dominated by perennial and annual grasses such as California oatgrass (*Danthonia californica)*, redtop (*Agrostis gigantica*), and softchess (*Bromus horeaceus*). Forbs such as buttercup (*Ranunculus*) and clover (*Trifolium*) were also present. Reed canary grass (*Phalaris arundinacea*) has become prevalent since the early 2000s and presumably is less palatable to elk [19,21]. The Davison meadow complex is surrounded by old-growth and second growth coastal redwood (*Sequoia sempervirens*) forest. Common conifers include Douglas fir (*Pseudotsuga menziesii*), Sitka spruce (*Picea sitchensis*), and western hemlock (*Tsuga heterophylla*). Dense canopy cover in the surrounding forest prevents many grasses and forbs from growing. Predators of elk are mountain lions (*Puma concolor*) and black bear (*Ursus americanus*). Neither predator appears to exert much top-down influence on elk population dynamics in the parks [19,20].

The Davison meadow complex covers 50 ha. Cattle grazing began in the late 1890s after forest was removed and meadow vegetation was established [19]. Cattle were the predominant herbivores that grazed the Davison meadow complex until 1991 when the area was purchased by the Save the Redwoods League and gifted to the National Park Service [21]. After 1991 cattle grazing ceased, and elk colonized and began grazing the meadow complex [19]. Subsequently there was a population irruption with a peak in abundance in 1997, a steady decline in abundance until 2006, and thereafter abundance steadily increased until the completion of this study in 2019 [19,21,31]. Two elk herds have used the Davison meadows. A herd is comprised of females, juveniles, and subadult males that form a cohesive group [32] that rarely coalesce with other elk herds [19]. The Davison herd has grazed the Davison meadow complex as its source of forage in winter across the length of the study [19]. The Levee herd, which usually occupied meadows to the south of the Davison complex, also grazed the southern part of the Davison meadow complex from 2007 to 2012. In 2016, another 10-ha cattle pasture, immediately south of the Davison meadow complex, became available to elk [22].

Black-tailed deer (*Odocoileus hemionus columbianus*) were the only other large herbivore in the Davison meadow complex. Deer, however, were scarce. Other herbivores included the California vole (*Microtus californicus*) and phytophagous insects.

### Vegetation data

Vegetation was measured each January between 2005 and 2019. Based on ostensible differences in vegetation and to ensure that all parts of the Davison meadow complex were measured, we carved up the Davison meadow complex into 7 sectors (Fig 1). Sectors ranged in size from 2–10 ha. We estimated forage biomass within 270 ¼ m2 plots (250 plots in 2005) located along transects that were randomly placed in each of the 7 sectors. The sectors grazed by herds across the 15 years of the study were horsebarn, A, B, and C, WPC, picnic, and south Davison (Fig 1). Vegetation in fish hatchery and cattle pasture sectors was not included in analyses because these two sectors began being grazed in 2016 when the Davison herd expanded its home range [22]. The number of transects within each sector, and the number of plots within each transect, was proportional to the size of the sectors. Each transect had 10–30 plots spaced 10 meters apart and transects were at least 20 meters apart. In each plot we estimated vegetation height to the nearest centimeter in 8 equidistant places. We also estimated coverage of palatable grasses, forbs, and shrubs using Daubenmire coverage classes: 0–5%, 6–25%, 26–50%, 51–75%, 76–95%, and 96–100% [33]. From 2005–2007, these same measurements were taken in 129 additional plots distributed throughout the 7 sectors. Vegetation within these plots was then clipped to ground level and sorted into palatable grasses, forbs, and shrubs. These samples were then dried for 48 hours at 60°C and weighed to measure dried biomass [19]. Following this, multiple regressions were estimated using average vegetation height within each plot and Daubenmire coverage classes to predict dried biomass of grasses (g ¼ m^2^) (*r*^2^ = 0.84, *F*7, 122 = 97.1, *P* < 0.001) and forbs (*r*^2^ = 0.33, *F*2, 93 = 24.9, *P* < 0.001) [19]. These regressions were used to estimate forage biomass within each plot in every year of the study.

### Weather and elk abundance

Precipitation can limit plant growth, but cold temperatures might also be influential. We measured average monthly low temperature and monthly precipitation (cm) in 3 months that were early in the growing season: October, November, and December. Weather in these 3 months were likely to influence forage biomass the following January [31,34]. Weather data was acquired from National Oceanic and Atmospheric Administration land-based weather stations. Initially, weather data was obtained from the Prairie Creek State Park station (station # 046498) located 3 km north of the Davison meadow complex. There were many months of missing data, however. We estimated these missing values from a regression that used data from a weather station 48km to the north near Crescent City, California, USA (station # 042147). Data from the Crescent City station predicted monthly precipitation (*r 2* = 0.61) and low temperature (*r*2 = 0.70) in the Davison meadow complex [21,31].

Previous work has shown that the time spent grazing by the Davison herd is directly proportional to food availability in the sectors of the Davison meadow complex [19,22], so it is likely that elk abundance is a useful index of herbivory and trampling across the Davison meadow complex. To estimate elk abundance, we conducted 10 population surveys each January. Each survey began at sunrise and lasted 1.75 hours. For each survey, we drove a pre-determined route through the Davison meadow complex and recorded the number of adult and subadult females, subadult males, and juveniles. The herds were habituated to people, and so all observations were carried out within 200 meters of elk using binoculars or the naked eye. We did not include adult males because they do not graze the Davison meadow complex to the same degree as female herds [35,36]. We used the highest count across the 10 surveys as our estimate of population abundance for each year. The high count was adequate to index abundance because detection probabilities of females were high (> 0.8) in the Davison meadow complex [19].

### Analyses

We conducted an initial analysis to determine what weather variables (precipitation, low temperature) had the largest influence on forage biomass and at what temporal scale the variables had the strongest influence. Precipitation was natural log transformed because a positive, asymptotic relationship with forage biomass was evident in scatterplots. Variables correlated with forage biomass were examined monthly for October to December, November–December, and October–December. We estimated forage biomass at the scale of Davison meadow complex because multiple studies have found that relationships between environmental factors are more pronounced at a larger scale [37–39]. This analysis indicated that low temperature at any monthly scale was not statistically significant nor did low temperature have the influence on forage biomass in the Davison meadow complex as did precipitation that fell between October and December (Supplementary material, S1 Table). In subsequent analyses we used precipitation summed across October through December as the weather variable. For convenience, hereafter we refer to October–December precipitation as precipitation.

We conducted analyses at two spatial scales, the Davison meadow complex and sector. At the scale of the Davison meadow complex, we estimated a general linear model with natural logarithm of precipitation, elk abundance, and a categorical predictor for expansion as predictors of forage biomass. Elk abundance was the abundance of the Davison herd plus the abundance of the Levee herd in the years 2007–2012, years that the Levee herd was detected in the Davison meadow complex. A preliminary analysis indicated that abundance of the Davison herd alone did not influence forage biomass (Supplementary material, S2 Table). Once the Davison elk herd expanded its range into the cattle pasture and fish hatchery sectors in 2016 it was possible that grazing pressure was reduced in the 7 remaining sectors. Forage biomass was the sum of the forage biomass in the seven sectors continually grazed across the 15 yearlong study. At the sector scale, we estimated 2 linear mixed effects models where forage biomass in ¼ m^2^ plots was the response variable [40]. One model had fixed predictors of precipitation (log transformed), elk abundance, and expansion. Elk abundance was scaled to have a mean of zero and standard deviation of one. The random factor was year which was categorically coded and had an intercept random effect. The other mixed effect model we estimated had the same fixed predictors but with more elaborate random effects considering sector. Again, year was a random factor with a random intercept as the effect. The other random factor was sector, and sector had intercept and slope random effects for both elk abundance and precipitation. Consequently, for the sector-specific regressions (abundance–forage biomass, precipitation–forage biomass) estimated by this mixed effect model, correlation coefficients were also estimated between intercepts and slopes coefficients, and between slope coefficients. Interpreting these correlations allowed us to assess if forage biomass in sectors responded, or not, to both elk abundance and precipitation. We conducted a likelihood ratio test between the two models to determine if substantial heterogeneity existed across sectors in relationships between abundance, precipitation, and forage biomass [40]. For the selected model we estimated 95% confidence intervals for all fixed and random effects using a parametric bootstrap approach from 1000 simulations.

## Results

**Fig 2:**
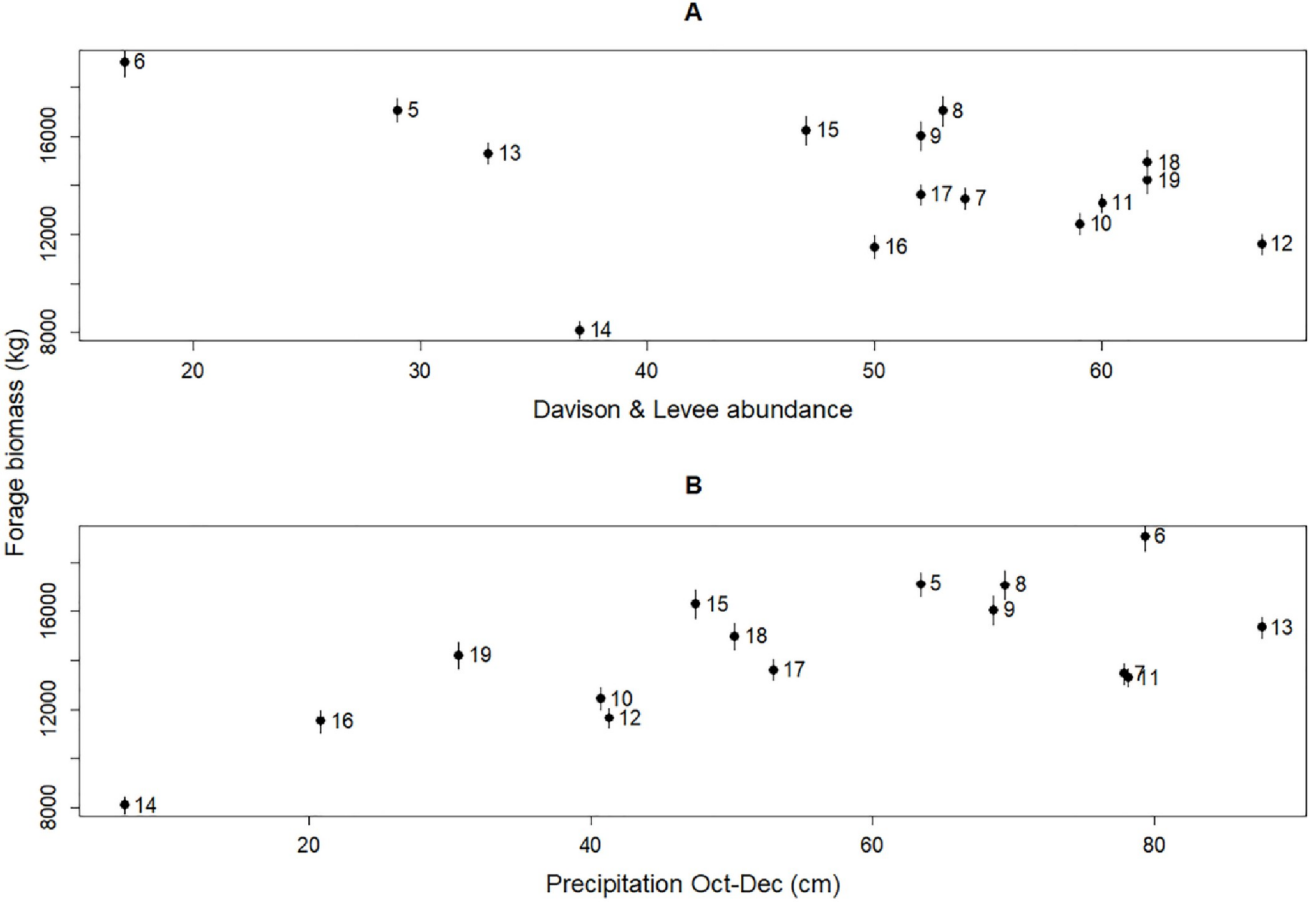
Scatterplots of elk abundance (A), precipitation (B), and forage biomass in the 7 sectors continually grazed in the Davison meadow complex (plus 1 standard error bars) across the 15 years of the study (2005–2019). Numbers next to symbols are the years (e.g., 5–2005, 19–2019).

From 2005 to 2019, forage biomass in the Davison meadow complex ranged from 8,257 (SE = 365) kg in 2014 to 19,432 (6,277) kg in 2006 and averaged 14,542 (138) kg. Elk abundance ranged from 17 individuals in 2006 to 67 individuals in 2012 and averaged 49 individuals. Precipitation from October through December ranged from 17.6 cm in 2014 to 222.4 cm in 2013 and averaged 137.9 cm. Elk abundance was negatively related and precipitation was positively related to forage biomass in the Davison meadow complex (Table 1, Fig 2A and 2B). Expansion, however, was not influential.

**Table 1:** Estimates, standard errors, and t-tests from a general linear model examining influences of elk abundance, October–December precipitation (natural log transformed), and expansion on forage biomass (kg) in the Davison meadow complex. Expansion was a categorical variable for years before (2005–2015, coded 0) and after (2016–2019, coded 1) the Davison herd expanded its home range. Forage biomass was the sum of biomass in the seven sectors (South Davison, A, B, C, Picnic, WPC, and Horsebarn) continuously grazed by Davison and Levee herds between 2005 and 2019. The model adjusted *r*^2^ was 0.67 and the residual standard error was 1567. One-tailed probability values are reported for abundance and precipitation as we expected abundance to be inversely, and precipitation to be positively related to forage biomass.

Coefficient	Estimate	Standard error	*t*	*P*
Intercept	5723.9	2961.5	1.93	0.0772
Abundance	-69.5	30.0	-2.32	0.0135
Precipitation	3104.1	637.1	4.87	0.0002
Expansion	1054.1	1001.4	1.05	0.3151

There was heterogeneity in forage biomass relationships at the spatial scale of the sector. The mixed effect model with only year as a random factor did not fit the data as well as the model with year and sector as random factors (*Χ*^*2*^ = 1053.6, *df* = 6, *P* < 0.001). In the selected model, the fixed predictor of elk abundance was not influential, but precipitation was positively related to sector forage biomass (Table 2). Expansion, again, was not influential. Standard deviation of the year intercept was noticeably less than the sector intercept and residual as evident by nonoverlapping confidence intervals. Abundance and precipitation slope random effects displayed variability as lower bounds of confidence intervals did not include 0. For abundance, slope variability indicates that the fixed effect coefficient, which is averaged across sector-specific coefficients, does not necessarily mean that abundance did not influence forage biomass in some sectors [40].

**Table 2:** Estimates and 95% confidence bounds of the fixed and random effects of a linear mixed-effects model summarizing forage biomass. Abundance was scaled to have a mean of zero and standard deviation of one and Oct–Dec Precipitation was natural logarithmic transformed. Reference category for expansion is years before (2006–2016) the Davison herd expanded into the fish hatchery and cattle pasture sectors. Year had only intercept random effects whereas abundance and precipitation had intercept and slope random effects.

**Fixed effects**				
Coefficient	Estimate	Lower bound	Upper bound
Intercept	1.39	-2.42	4.91
Abundance	-0.45	-1.07	0.17
Oct–Dec precipitation	1.36	0.24	2.44
Expansion	0.53	-0.44	1.47
**Random effects**				
Attribute	Standard deviation	Lower bound	Upper bound
Intercept—year	0.76	0.43	1.10
Intercept—sector	3.52	1.35	5.78
Slope—abundance	0.57	0.23	0.91
Slope—precipitation	1.19	0.47	1.90
Residual	3.57	3.49	3.65
	Correlation		
Intercept and slope–abundance	0.82	0.15	1.00
Intercept and slope–precipitation	-0.93	-0.99	-0.62
Slopes–abundance and precipitation	-0.87	-0.99	-0.37

**Fig 3:**
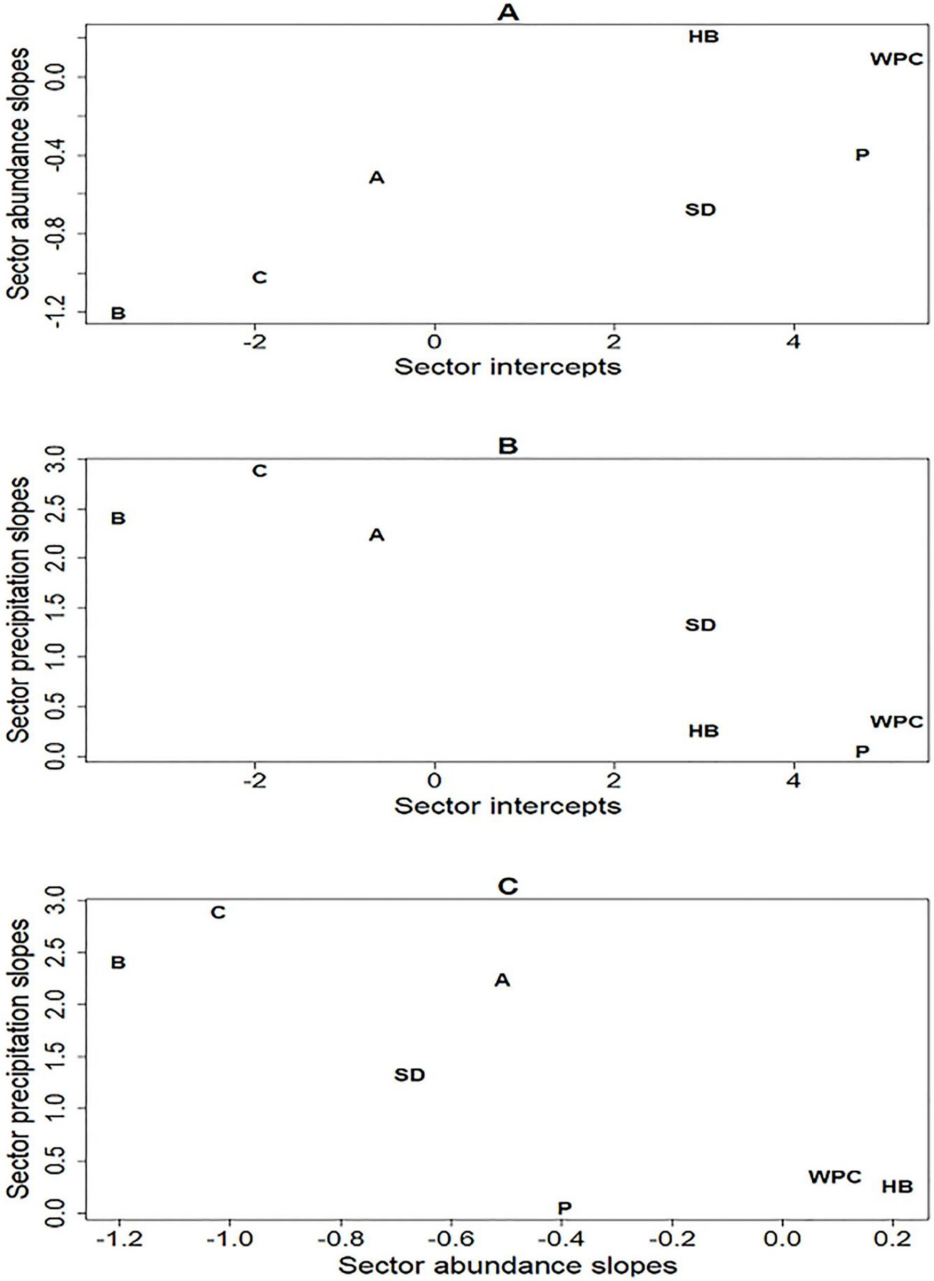
Scatterplots of pairwise comparisons of sector-specific regression coefficients estimated by the linear mixed effect model in the 7 sectors continually grazed across the 15 years of the study. In A are intercepts and slope coefficients of relationships between elk abundance and forage biomass. In B are intercepts and slope coefficients of relationships between precipitation from October to December and forage biomass. In C are slope coefficients of relationships between elk abundance and forage biomass, and precipitation and forage biomass. Letters or initials identify sectors: HB is horse barn, P is picnic, and SD is south Davison.

The nature of heterogeneity in forage biomass across sectors was revealed by the correlations between sector-specific regression coefficients (Table 2). Intercepts and slopes were positively correlated for the abundance predictor but negatively associated for precipitation as 95% confidence intervals for these correlation coefficients excluded 0. For abundance, sectors with more forage biomass, as indicated by intercepts of 0 for scaled abundance, also had more negative slopes (Fig 3A). In sectors such as B and C, forage biomass appeared to be more responsive to more intense grazing pressure as indexed by elk abundance. On the other hand, in WPC and horse barn forage biomass responded little to elk abundance because slopes were near 0. For precipitation, sectors such as B and C with large positive slopes had small intercepts indicating forage biomass responded to moisture more than in WPC and horse barn, for example, where intercepts were larger, but slope estimates were near 0 (Fig 3B). Responses to elk abundance and precipitation were similar across sectors as abundance and precipitation slopes were inversely correlated (Fig 3C). For example, in sectors B and C abundance coefficients were small because of the inverse relationship between abundance and forage biomass but precipitation coefficients were large because more precipitation led to more forage biomass. Whereas sectors such as WPC and horse barn, where forage biomass did not respond to either predictor, sector-specific regressions had slopes close to 0. To summarize, in some sectors forage biomass was apparently unaffected by elk abundance and precipitation, whereas in other sectors forage biomass declined with increasing elk abundance and increased with more precipitation (Supplementary material, S1 Fig).

**Fig 4:**
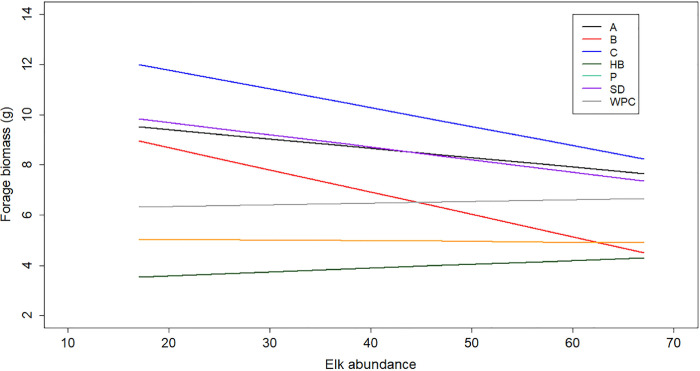
Scatterplot of estimated sector-specific regressions between elk abundance and forage biomass (1/4 m^2^) from the linear mixed effect model. These regressions were estimated using mean precipitation (54.31 cm) and during the years of no herd expansion. Sector labels are the same as in Figs 1 and 3 captions.

Controlling for precipitation, it was possible to show that forage biomass declined with increasing elk abundance across sectors (Fig 4). Sectors where forage biomass declined with increasing forage abundance approached forage biomass estimates in sectors where forage appeared to be unaffected by elk abundance.

## Discussion

As far as we know our study is the first to detect a density associated response of a large grazer to biomass of its food supply measured at the spatial scale inhabited by the free ranging population. On the other hand, detecting an inverse relationship between herbivore abundance and biomass of its food supply is expected. The population dynamics of both the Davison and Levee herd are governed by strong density dependence, and bottom-up processes likely exert a greater impact than top-down influences [19,20,22,31]. Nonetheless, the density associated relationship might not have been revealed without considering precipitation which varied across the 15 years of study. A clear example is forage biomass in 2014. In that year there was intermediate not high elk abundance, but precipitation was the lowest measured. Coinciding with low precipitation was the lowest forage biomass measured in the Davison meadow complex across the 15 years of the study. In free-ranging populations, factors influencing large, grazing ungulate food supplies can be complicated.

Another bit of evidence indicating that elk abundance exerted an immediate influence on Davison meadow forage abundance was that elk abundance measured by the Davison herd alone was not influential. Between 2007 and 2012 the Levee herd, which usually inhabited meadows to the south also grazed the Davison meadow complex [19]. During these years, the two herds were similar in abundance and lumped together these abundances were as high as what has been documented across 25 years of study [19,20]. The presumed intense grazing pressure from herbivory and trampling exerted by high elk abundance negatively impacted forage biomass in the Davison meadow complex.

Precipitation displayed a positive, asymptotic relationship with forage biomass. Temperatures in winter might partly explain why there was a nonlinear relationship. Cooler temperatures early in the plant growing season can limit the maximum rate at which plants grow by suppressing soil microbial communities that mineralize nitrogen for plants. In California annual grasslands soil nitrogen and phosphorous are lower in the early winter than in the late winter and early spring [41,42]. Cooler temperatures in early winter monthS probably slowed the rates at which microbial communities were able to assimilate soil nutrients which probably reduced the rate of nutrient availability to plants. In years with abundant precipitation from October to December, plants probably could not capitalize on the increased moisture.

Examining influences on food supply at the spatial scale of the population did not offer as complex a picture as what was found at the spatial scale of parts of the area used by the elk population, sectors. Some sectors had forage biomasses that were unaffected by elk abundance and precipitation and in other sectors forage biomass responded to these two predictors as was found at the scale of the meadow complex. Heterogeneity in response to elk abundance and precipitation across sectors is unlikely to result from uneven grazing pressure across sectors. The Davison and Levee herds were cohesive groups so all individuals in these populations grazed in close spatial proximity. When elk foraging is not affected by inclement weather or lethal risks, the foraging time of herds in sectors should be dictated by the amount of forage [39,43]. Indeed, grazing time in sectors by herds was proportional to forage biomass [19,22].

It is likely that variation in soil characteristics across sectors had a role in impacting plant productivity and forage biomass. The sectors that showed no detectable influence from elk abundance and precipitation (horsebarn, picnic, WPC) had a history of anthropogenic disturbances. The horse barn was used to store heavy equipment within the last 30 years, and part of the area was covered by asphalt. Likewise, WPC was the site of the Davison family homestead. The picnic area was a restored logging deck that had large asphalt pads in the past 30 years [19]. Gravel deposition and asphalt can degrade soils [44] and negatively impact native microbial communities [45].

Because plants in some sectors responded little to precipitation and elk abundance, annual fluctuations in forage biomass at the scale of the Davison meadows is mostly driven by forage biomass in the productive sectors. It seems likely, as such, that elk population dynamics are also tied to the sectors where plants responded to elk abundance and precipitation more so than in sectors where plants were less responsive. Because we had information on elk spatial grazing patterns and spatial heterogeneity in forage biomass, we were able to predict that across sectors heterogeneity in forage biomass would decline with increasing elk abundance averaging for precipitation [4]. Grazing pressure of a herd in a sector was constant relative to available forage biomass, not size of a sector or forage biomass in adjacent sectors [19]. At low abundance there was, on average, greater per capita forage biomass at the spatial scale of a bite station in sectors then at abundances closer to *K* carrying capacity [46]. The density associated decline in spatial heterogeneity of forage biomass might reduce meadow complexity and resilience [28]. Complexity and resilience of the Davison meadows plant communities might be particularly impacted by elk grazing because the Davison herd presumably had already impacted meadow vegetation when the herd irrupted in the 1990s [21,31].

Examination of forage biomass at the scale of the sector was helpful in explaining why expansion of the Davison herd into new sectors was not influential. A herd was a group comprised of the same elk that repeatedly foraged in sectors at the scale of weeks [19,22]. Consequently, grazing pressure should be reduced in any given sector as the entire food supply is enlarged via the addition of new sectors. As a result, there should be reduced time grazing in every sector. But this scenario assumes that the food supply in any given sector is affected by elk grazing and trampling alone, and that shortly before and after the expansion occurred elk abundance changed little. Assumptions that were unrealistic because elk abundance increased notably after expansion and the influence of precipitation varied independently of elk abundance [20,22].

Elk abundance negatively affected forage biomass but across our analyses at two spatial scales the effect of this predictor was not detected as consistently as was precipitation. The fixed effect for elk abundance was not substantial. Herbivore optimization might be one possibility. If elk grazing and trampling at low and intermediate abundances stimulated net aboveground primary productivity, which seems likely in our moist study area, then it is feasible that a variable measuring forage state such as biomass would show little variation with forage biomass across low to intermediate elk abundances. As we only measured forage state, biomass, and not net aboveground primary productivity we could not evaluate if herbivore optimization had occurred. A further challenge to detecting herbivore optimization from forage state is that elevated net aboveground primary productivity can display considerable variability at the large spatial scales occupied by elk [10]. If a study of ungulate grazing is not designed to detect herbivore optimization, which would include measuring aboveground net primary productivity, then it will be difficult to detect when only forage state is measured. Another possibility is that per capita food intake might have varied across years from carry over effects that occurred the previous summer and fall when forage quality was low, and it might have been low for an extended period [47–49]. Per capita food intake in such a setting might then increase when green feed becomes available in late fall [19,38]. An elevated, per capita food intake would not necessarily be reflected in elk abundance alone. If we had been able to measure per capita food intake in addition to elk abundance we might have more accurately measured elk grazing pressure.

Carving up Davison meadow complex into sectors based on our perception of heterogeneity in forage probably does not mimic how elk might perceive heterogeneity in their food supply. Our findings about sector heterogeneity in forage biomass and the influences of elk abundance and precipitation, therefore, can be viewed suspiciously. We contend that our findings and conclusions offer insights. Elk abundance should display an inverse effect and precipitation a positive effect on biomass of grazing tolerant, herbaceous plants. Moreover, there is theory to expect longer time foraging in places with more forage which also has support from empirical study [39,43]. How we carved up Davison meadow complex in sectors might not capture the heterogeneity in forage biomass as perceived by elk, but the patterns we detected should capture how elk are likely to respond to perceived heterogeneity in biomass of the food supply.

The stability of large grazing herbivores with the food supply has often focused on highly managed, in other words controlled, domestic livestock grazing in discrete and confined areas [9,50,51]. Nevertheless, stable grazing systems seem possible for free ranging elk populations. There are empirical examples of free ranging elk populations under bottom-up influences reaching a dynamic equilibrium with the food supply, or *K* carrying capacity at temporal scale of decades [52,53]. In our study it is plausible that a dynamic equilibrium between elk population abundance and meadow food supplies is possible because there was an inverse relationship between herd abundance and biomass of the food supply and low precipitation that substantially reduces plant growth was infrequent. But across the 15 years of this study a dynamic equilibrium between elk and the food supply probably has been delayed because the food supply has enlarged with the expansion of the Davison herds home range [20,22]. Detecting relationships between free ranging ungulate populations and biomass of their food supplies is not straightforward.

### Supplementary information

Two tables (S1 and S2 Tables) containing correlation coefficients between precipitation and temperature, and parameter estimates of the general linear model examining the influences of Davison herd abundance, precipitation, and expansion on forage biomass. Also, one S1 Fig shows sector-specific scatterplots of data and relationships estimating forage biomass. An Excel file in CSV format has the forage biomass data.

## Supporting information

S1 Fig Scatterplots of sector data and relationships for each predictor, scaled elk abundance and natural logarithm of precipitation from October to December with forage biomass (g^.^ ¼ m^2^). Sector labels are the same as described in Fig 3 caption.
(DOCX)

S1 Table Pearson’s correlation coefficients of precipitation and low temperature with forage biomass in Davison meadow complex from October to December, 2005–2019. Precipitation was the total for each month or months. Low temperature was averaged across days of each month or months. Sample size for each correlation was 15. Correlation coefficients ≥ 0.66 were statistically significant (*P* < 0.05). Bold font indicates strongest correlation.
(DOCX)

S2 Table Estimates, standard errors, and t-tests from a general linear model examining influences of Davison herd abundance, October–December precipitation (natural log transformed), and expansion on forage biomass (kg) in the Davison meadow complex. Expansion was a categorical variable for years before (2005–2015, coded 0) and after (2016–2019, coded 1) the Davison herd expanded its home range. Forage biomass was the sum of biomass in the seven sectors (South Davison, A, B, C, Picnic, WPC, and Horsebarn) continuously grazed by the Davison herd between 2005 and 2019. The model adjusted *r*^2^ was 0.49 and the residual standard error was 1961. One-tailed probability values are reported for abundance and precipitation as we expected abundance to be inversely and precipitation positively related to forage biomass.
(DOCX)

S1 Data (XLSX)
