# Supplementary material for: Influences on food supply from elk abundance and precipitation early in the growing season
Source: PLoS One. 2022 Mar 11;17(3):e0264941. doi: 10.1371/journal.pone.0264941 (PMC8916677; doi:10.1371/journal.pone.0264941)

Figure S1. Scatterplots of sector data and relationships for each predictor, scaled elk abundance and natural logarithm of precipitation from October to December with forage biomass (g ^.^ ¼ m^2^). Sector labels are the same as described in figure 3 caption.


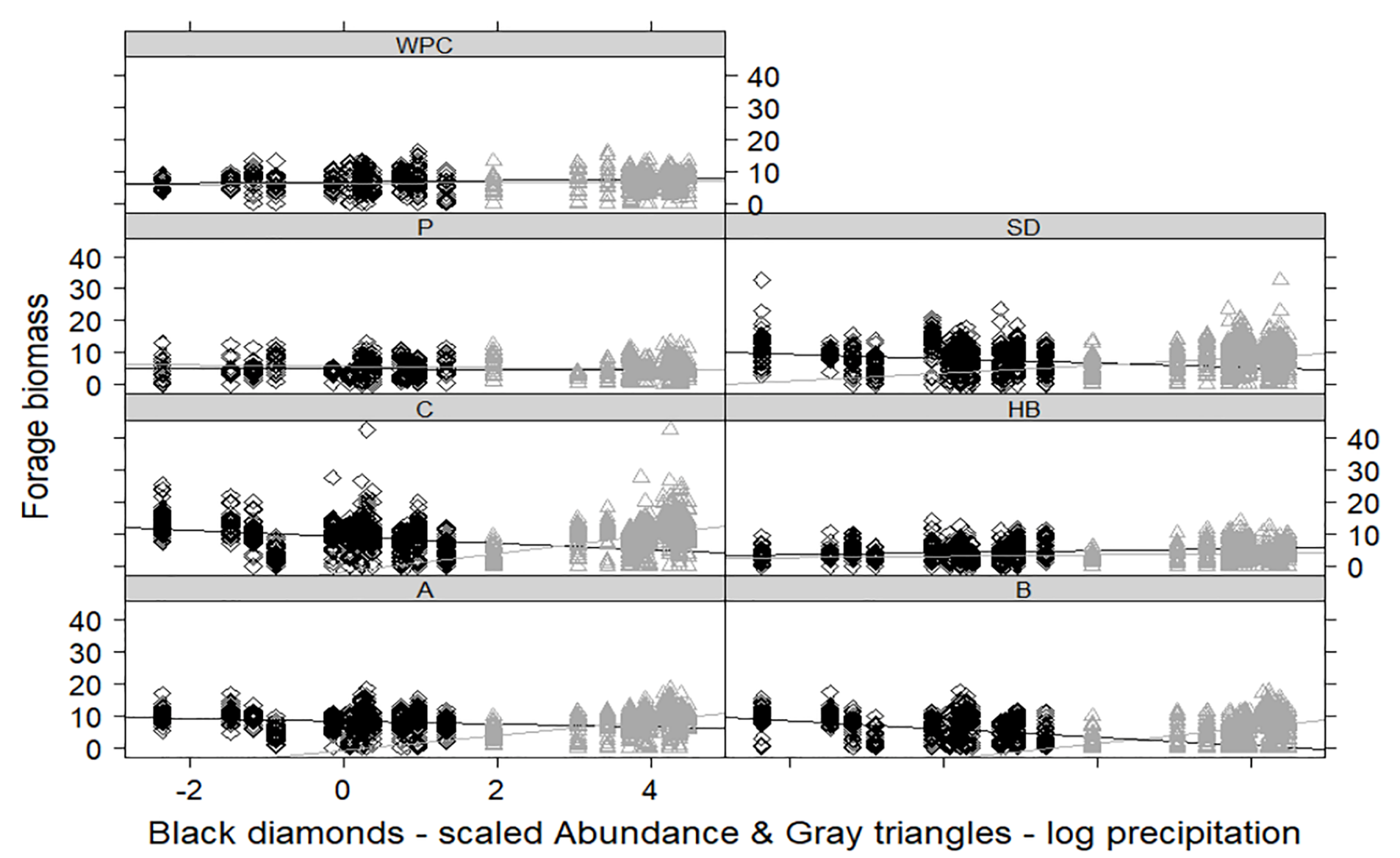

Supplement: S1 Fig — Sector labels are the same as described in Fig 3 caption. (DOCX) [file pone.0264941.s001.docx]
