# Supplementary material for: Influences on food supply from elk abundance and precipitation early in the growing season
Source: PLoS One. 2022 Mar 11;17(3):e0264941. doi: 10.1371/journal.pone.0264941 (PMC8916677; doi:10.1371/journal.pone.0264941)
Supplement: S1 Table — Precipitation was the total for each month or months. Low temperature was averaged across days of each month or months. Sample size for each correlation was 15. Correlation coefficients ≥ 0.66 were statistically significant (P < 0.05). Bold font indicates strongest correlation. (DOCX) [file pone.0264941.s002.docx]

Table S1**.** Pearson’s correlation coefficients of precipitation and low temperature with forage biomass in Davison meadow complex from October to December, 2005 – 2019. Precipitation was the total for each month or months. Low temperature was averaged across days of each month or months. Sample size for each correlation was 15. Correlation coefficients > 0.66 were statistically significant (*P* < 0.05). Bold font indicates strongest correlation.

|  | | |
| --- | --- | --- |
| Variable | Precipitation | Temperature |
| October | 0.66 | 0.21 |
| November | 0.42 | 0.10 |
| December | 0.70 | 0.44 |
| November – December | 0.72 | 0.35 |
| October – December | **0.77** | 0.35 |
