# Supplementary material for: Influences on food supply from elk abundance and precipitation early in the growing season
Source: PLoS One. 2022 Mar 11;17(3):e0264941. doi: 10.1371/journal.pone.0264941 (PMC8916677; doi:10.1371/journal.pone.0264941)
Supplement: S2 Table — Expansion was a categorical variable for years before (2005–2015, coded 0) and after (2016–2019, coded 1) the Davison herd expanded its home range. Forage biomass was the sum of biomass in the seven sectors (South Davison, A, B, C, Picnic, WPC, and Horsebarn) continuously grazed by the Davison herd between 2005 and 2019. The model adjusted r2 was 0.49 and the residual standard error was 1961. One-tailed probability values are reported for abundance and precipitation as we expected abundance to be inversely and precipitation positively related to forage biomass. (DOCX) [file pone.0264941.s003.docx]

Table S2. Estimates, standard errors, and t-tests from a general linear model examining influences of Davison herd abundance, October – December precipitation (natural log transformed), and expansion on forage biomass (kg) in the Davison meadow complex. Expansion was a categorical variable for years before (2005 – 2015, coded 0) and after (2016 – 2019, coded 1) the Davison herd expanded its home range. Forage biomass was the sum of biomass in the seven sectors (South Davison, A, B, C, Picnic, WPC, and Horsebarn) continuously grazed by the Davison herd between 2005 and 2019. The model adjusted *r*^2^ was 0.49 and the residual standard error was 1961. One-tailed probability values are reported for abundance and precipitation as we expected abundance to be inversely and precipitation positively related to forage biomass.

| Coefficient | Estimate | Standard error | *t* | *P* |
| --- | --- | --- | --- | --- |
| Intercept | 377.02 | 4748.79 | 0.079 | 0.9381 |
| Abundance | 31.03 | 75.63 | 0.41 | 0.6894 |
| Precipitation | 3347.08 | 867.57 | 3.86 | 0.0027 |
| Expansion | -561.24 | 2215.41 | -0.253 | 0.8047 |
